# Hidden Blood Loss in Full-Endoscopic Lumbar Decompression Compared with Biportal Endoscopic and Open Microscopic Surgery for Single-Segment Lumbar Stenosis

**DOI:** 10.3390/jcm15103926

**Published:** 2026-05-20

**Authors:** Sung Cheol Park, Yongjung Kim, Sang Soo Eun, Hee Jung Son

**Affiliations:** 1 Department of Orthopaedic Surgery, Spine Endoscopy Center, Bumin Hospital Seoul, Seoul 07590, Republic of Korea; osspinepark@gmail.com (S.C.P.); yjkimmd85@gmail.com (Y.K.); erupt0123@naver.com (S.S.E.); 2 Department of Orthopedic Surgery, Nowon Eulji Medical Center, Eulji University School of Medicine, Seoul 03080, Republic of Korea

**Keywords:** lumbar spinal stenosis, endoscopic spine surgery, full-endoscopic spine surgery, biportal endoscopic spine surgery, unilateral laminotomy with bilateral decompression, hidden blood loss, minimally invasive spine surgery

## Abstract

**Background/Objectives**: Accurate estimation of intraoperative blood loss in endoscopic spine surgery remains challenging because of continuous saline irrigation and blood infiltration into surrounding soft tissues and potential dead spaces. Hidden blood loss (HBL), resulting from extravasation into tissue compartments or hemolysis, may substantially increase total blood loss (TBL) and contribute to postoperative bleeding-related complications. This study aimed to compare HBL in full-endoscopic unilateral laminotomy with bilateral decompression (FE-ULBD) with that in biportal endoscopic ULBD (BE-ULBD) and open microscopic ULBD (OM-ULBD). **Methods**: A retrospective analysis was conducted of patients who underwent single-level FE-ULBD, BE-ULBD, or OM-ULBD for lumbar spinal stenosis (LSS) at a single institution. Data on perioperative characteristics, laboratory parameters, perioperative blood loss (TBL, HBL, and visible blood loss), and clinical outcomes were collected and compared. Univariate linear regression analyses were performed to identify factors associated with HBL in the FE-ULBD group. **Results**: A total of 93 patients were included, comprising 34 in the FE-ULBD group, 32 in the BE-ULBD group, and 27 in the OM-ULBD group. The FE-ULBD group demonstrated significantly lower TBL than both the BE-ULBD and OM-ULBD groups (493.20 ± 183.46 vs. 675.97 ± 192.02 vs. 822.94 ± 424.11 mL, *p* = 0.001 and *p* = 0.002, respectively). HBL in the FE-ULBD group was significantly lower than in the BE-ULBD group (390.48 [268.32–506.91] vs. 513.29 [437.96–633.36] mL, *p* = 0.012) and was numerically lower than in the OM-ULBD group without statistical significance (390.48 [268.32–506.91] vs. 516.38 [316.41–710.68] mL, *p* = 0.081). Male sex was the only variable significantly associated with increased HBL in the FE-ULBD group. **Conclusions**: FE-ULBD showed significantly lower TBL than BE-ULBD and OM-ULBD, and lower HBL than BE-ULBD. FE-ULBD may represent a feasible surgical option for single-level LSS, with the potential advantage of reduced perioperative blood loss while maintaining comparable clinical outcomes.

## 1. Introduction

Lumbar spinal stenosis (LSS) represents one of the most common degenerative conditions of the lumbar spine in older adults [1]. The condition typically presents with low back pain, radicular pain in the lower extremities, and neurogenic claudication, leading to impaired ambulation and reduced quality of life [2,3]. Surgical intervention may be considered for patients who remain refractory to adequate conservative management or who develop neurological deficits, including motor weakness and bladder or bowel dysfunction [4,5]. Decompression surgery, the standard operative treatment for LSS, has demonstrated effectiveness in pain relief and functional improvement [6]. Among the various decompression techniques, unilateral laminotomy with bilateral decompression (ULBD) has gained popularity, as it minimizes injury to posterior ligamentous structures and thereby reduces the risk of iatrogenic segmental instability [5].

As the population ages, minimally invasive spine surgery has gained increasing acceptance for the treatment of patients with multiple comorbidities and significant frailty [7,8]. In particular, endoscopic spine surgery (ESS) is recognized for minimizing trauma to anatomical structures and reducing overall surgical burden, thereby resulting in less postoperative pain and faster recovery [9]. Two distinct endoscopic approaches are currently in use: full-endoscopic spine surgery (FESS) and biportal endoscopic spine surgery (BESS). Although both techniques share the same surgical objective and indications, they differ in their operative corridors [5]. In FESS, both the endoscope and surgical instruments are introduced through a working sleeve via a single skin incision. BESS, in contrast, requires two separate skin incisions: one for the viewing portal and one for the working portal. In the context of decompressive surgery for LSS, both approaches have been applied as ULBD procedures—full-endoscopic ULBD (FE-ULBD) and biportal endoscopic ULBD (BE-ULBD)—and have demonstrated favorable clinical and radiological outcomes [5,10].

Although ESS is generally associated with lower intraoperative blood loss (IBL), accurately estimating IBL remains challenging because of continuous saline irrigation and blood infiltration into surrounding soft tissues and potential dead spaces [11,12]. Hidden blood loss (HBL), resulting from extravasation into tissue compartments or hemolysis, may substantially increase total blood loss (TBL) and contribute to postoperative bleeding-related complications [3,4,13]. Despite several previous studies examining HBL in ESS, to the best of our knowledge, no study has evaluated HBL in full-endoscopic lumbar decompressive surgery performed via the interlaminar approach, such as FE-ULBD. Therefore, this study aimed to assess HBL in FE-ULBD in comparison with BE-ULBD and open microscopic ULBD (OM-ULBD), and to identify associated risk factors.

## 2. Materials and Methods

### 2.1. Study Patients

This retrospective study received approval from the institutional review board of our institution, with a waiver of informed consent granted because of the study design. Medical records were retrospectively reviewed for patients who underwent single-level FE-ULBD, BE-ULBD, or OM-ULBD for lumbar central canal and/or lateral recess stenosis between May 2023 and March 2025 at a single institution. ULBD was indicated for central stenosis in patients without spondylolisthesis or in those with Grade 1 spondylolisthesis without segmental instability. Patients with concurrent segmental instability or foraminal stenosis underwent alternative surgical procedures, such as fusion surgery, instead of ULBD. Eligible patients presented with radiating pain in the lower extremities and/or neurogenic intermittent claudication that was unresponsive to at least six weeks of conservative treatment or demonstrated neurological deterioration. Exclusion criteria included a history of surgery at the same spinal level, spinal infection, concomitant discectomy, or incomplete medical record documentation.

A total of 105 patients were initially identified. Patients with prior surgery at the same level (*n* = 3), infection (*n* = 1), concomitant discectomy (*n* = 2), or incomplete documentation (*n* = 6) were excluded, resulting in a final cohort of 93 patients. Of these, 34 patients underwent FE-ULBD, 32 underwent BE-ULBD, and 27 underwent OM-ULBD.

### 2.2. Surgical Technique

All procedures were performed by four orthopedic spine surgeons at a single institution. The choice of surgical technique—FE-ULBD, BE-ULBD, or OM-ULBD—was determined by individual surgeons’ preference following discussion with the patients. The operative side was determined according to symptom laterality and surgeon preference. Antithrombotic agents were routinely discontinued for at least 5 days prior to surgery in all patients according to our institutional protocol.

Patients were positioned prone on a Wilson frame under general anesthesia, and the operative level was confirmed using C-arm fluoroscopy. For FE-ULBD, a 9 mm skin incision was made targeting the caudal border of the upper lamina. Blunt dissection was performed with a dilator, followed by insertion of a working sleeve with the bevel oriented medially. A 20° endoscope (VERTEBRIS Stenosis Endoscopic System, 9.3 × 7.4 mm; RIWOspine GmbH, Knittlingen, Germany) was introduced through the working sleeve.

After exposure of the inferior border of the upper lamina using an electrocautery probe and a micro-rongeur, ipsilateral partial hemilaminectomy was performed using an endoscopic high-speed burr and a Kerrison punch. Medial facetectomy was then carried out to achieve bony decompression extending to the medial border of the lower pedicle. Following ipsilateral laminectomy, undercutting of the spinous process was performed to decompress the contralateral side. Partial laminectomy on the contralateral side was completed using an endoscopic high-speed burr and a Kerrison punch, followed by contralateral medial facetectomy until the medial border of the contralateral lower pedicle was reached. After completion of bony decompression, the ligamentum flavum was removed en bloc. Adequate neural decompression was confirmed by restoration of dural pulsation and complete visualization of the bilateral traversing nerve roots. Hemostasis was achieved using a gelatin–thrombin matrix hemostatic agent and radiofrequency cautery.

For BE-ULBD, two separate skin incisions were made: one for the viewing portal and the other for the working portal. Limited muscle detachment from the lamina was performed using a narrow Cobb elevator to create an adequate working space. A 4 mm, 30° endoscope was introduced through the viewing portal, while surgical instruments were inserted through the working portal. The ULBD procedure was then performed in the same manner as described above.

For OM-ULBD, a midline incision was made, followed by subperiosteal dissection of the paraspinal muscles to expose the lamina. A Caspar lumbar retractor was placed, and the procedure was performed under microscopic visualization. Subsequent decompression was carried out using the same surgical technique as described above.

A closed suction drain was placed in all patients before wound closure and maintained under negative pressure. The drain was removed when output was less than 30 mL over a 24 h period. Tranexamic acid was not administered in any patient.

### 2.3. Data Collection

**Figure 1:**
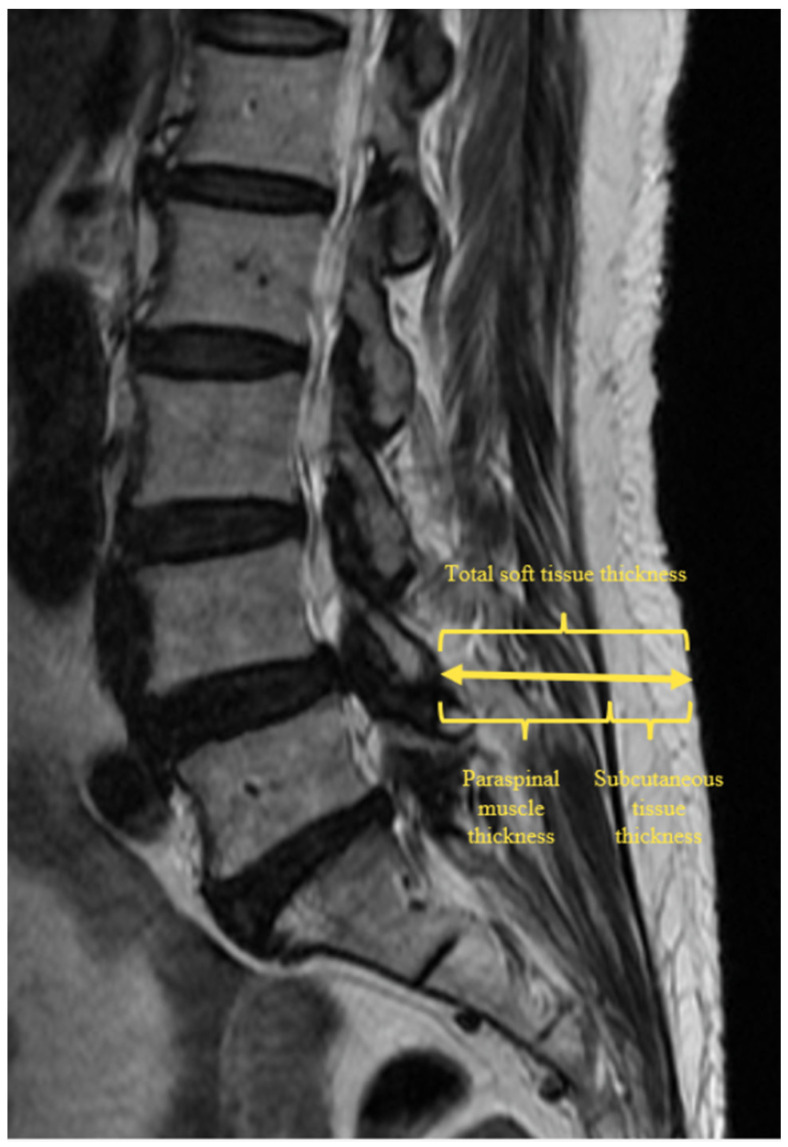
Measurements of total soft tissue, paraspinal muscle, and subcutaneous tissue thickness at the index level on parasagittal MRI of the operative side. MRI, magnetic resonance imaging.

We collected data on demographics (age, sex, body weight, height, body mass index); comorbidities (liver disease, antithrombotic medication, American Society of Anesthesiologists physical status classification system score); smoking status; operative details (surgical methods, operative level, operative side, operative time, IBL); degree of central canal stenosis, presence of spondylolisthesis; clinical outcomes (visual analogue scale [VAS], modified Macnab criteria); and perioperative complications. The degree of central canal stenosis was assessed using the Lee grading system [14]. IBL was measured by the anesthesiologist and circulating nurse based on the volume in the suction canister minus the amount of irrigation fluid used, with additional estimation from the weight of blood-tinged gauzes [11]. To assess coagulation status, preoperative platelet count, prothrombin time, international normalized ratio, and activated partial thromboplastin time were recorded. Preoperative and postoperative hemoglobin and hematocrit (Hct) levels were collected to estimate perioperative blood loss. Total soft-tissue thickness, paraspinal muscle thickness, and subcutaneous tissue thickness were measured on parasagittal magnetic resonance images of the operative side (Figure 1).

### 2.4. Calculation of Hidden Blood Loss

Patient blood volume (PBV) was calculated using the Nadler formula [15]:

PBV (L) = k_1_ × height (m)^3^ + k_2_ × weight (kg) + k_3_

where k_1_ = 0.3669, k_2_ = 0.03219, and k_3_ = 0.6041 for males; and k_1_ = 0.3561, k_2_ = 0.03308, and k_3_ = 0.1833 for females.

TBL was calculated using the Gross formula [16]:

TBL (L) = PBV × (Hct_pre_ − Hct_post_)/Hct_ave_

where Hct_pre_ represents the preoperative Hct measured closest to the date of surgery, Hct_post_ represents the lowest postoperative Hct during the hospital stay, and Hct_ave_ is the average of Hct_pre_ and Hct_post_.

TBL consists of visible blood loss (VBL) and HBL. VBL was defined as the sum of IBL and postoperative drainage volume. HBL was therefore calculated as follows:

HBL = TBL − VBL

### 2.5. Statistical Analysis

Normality was assessed using the Shapiro–Wilk test, and homogeneity of variances was evaluated using Levene’s test. For normally distributed variables, data were expressed as mean ± standard deviation, and comparisons among the three groups were performed using one-way analysis of variance (ANOVA). When both normality and homogeneity of variances were satisfied, Tukey’s post hoc test was used for pairwise comparisons. If the assumption of equal variances was violated, Welch’s ANOVA was applied, followed by Games–Howell post hoc tests. For variables that did not meet the normality assumption, data were expressed as median (interquartile range [IQR]), and group comparisons were performed using the Kruskal–Wallis test, followed by post hoc pairwise comparisons using Mann–Whitney U tests with Bonferroni correction. Categorical variables were compared using the chi-square test or Fisher’s exact test, as appropriate. For ordinal variables, the chi-square test for trend was applied. Comparison of VAS scores for radicular leg pain over time among groups was examined using repeated measures ANOVA (RM-ANOVA). Paired t-tests were applied for within-group comparisons between preoperative and 6-month postoperative VAS scores for radicular leg pain. Modified Macnab criteria were classified as favorable (excellent or good) or unfavorable (fair or poor) and were compared among groups using the cI confirm.hi-square test. Univariate linear regression analyses were performed to evaluate the associations between HBL in the FE-ULBD group and relevant independent variables. Given the relatively small sample size and the potential risk of overfitting, multiple linear regression analysis was not performed. All statistical analyses were performed using SPSS Statistics, version 25.0 (IBM Corp., Armonk, NY, USA). A *p*-value < 0.05 was considered statistically significant.

## 3. Results

Comparison of baseline characteristics, including demographic variables, operative details (operative level and side), degree of central canal stenosis, presence of spondylolisthesis, and comorbidities, revealed no significant differences among the three groups (Table 1). A comparison of perioperative data is presented in Table 2. No significant differences were observed in preoperative laboratory parameters, including hemoglobin, Hct, and coagulation profiles. Perioperative blood loss parameters, including TBL, VBL, and HBL, differed significantly among the groups (*p* < 0.001, *p* = 0.001, and *p* = 0.011, respectively). Post hoc analyses demonstrated that the FE-ULBD group had significantly lower TBL (493.20 ± 183.46 mL) than both the BE-ULBD (675.97 ± 192.02 mL, *p* = 0.001) and OM-ULBD groups (822.94 ± 424.11 mL, *p* = 0.002) (Table 3). No significant difference in TBL was observed between the BE-ULBD and OM-ULBD groups (*p* = 0.234). The FE-ULBD group showed significantly lower HBL than the BE-ULBD group (390.48 [268.32–506.91] vs. 513.29 [437.96–633.36] mL, *p* = 0.012). No significant differences were observed between the FE-ULBD and OM-ULBD groups (390.48 [268.32–506.91] vs. 516.38 [316.41–710.68] mL, *p* = 0.081) or between the BE-ULBD and OM-ULBD groups (*p* = 1.000). No patients required perioperative blood transfusion.

**Figure 2:**
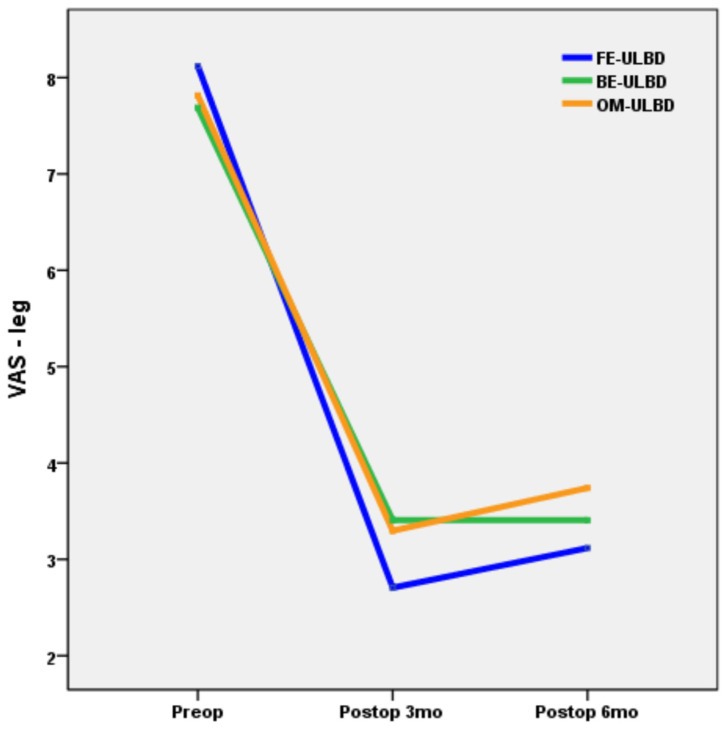
Comparison of VAS scores for leg pain among groups. VAS, visual analogue scale; FE-ULBD, full-endoscopic unilateral laminotomy with bilateral decompression; BE-ULBD, biportal endoscopic ULBD; OM-ULBD, open microscopic ULBD.

Table 4 compares clinical outcomes and perioperative complications among groups. RM-ANOVA showed no significant group-by-time interaction in VAS scores for radicular leg pain (*p* = 0.151), and no significant differences among groups (*p* = 0.727) (Figure 2). Paired t-test analysis demonstrated significant improvement in VAS scores for radicular leg pain at 6 months postoperatively compared with preoperative values in all groups (*p* < 0.001). There was no significant difference in the modified Macnab criteria (*p* = 0.344). The overall perioperative complication rates were comparable (*p* = 0.340). In the FE-ULBD group, two cases of dural tear and one case of root injury occurred. One case of hematoma formation and one case of dural tear were noted in the BE-ULBD group. In the OM-ULBD group, three cases of dural tear, one case of hematoma, and one case of wound problem were identified. All dural tears were managed intraoperatively with a fibrin sealant patch, and no case required reoperation or conversion to open surgery from ESS. Patients with hematoma were symptomatic and required surgical evacuation. The patient with root injury presented with persistent neuropathic pain without motor weakness.

Univariate analyses revealed male sex as the only variable significantly associated with increased HBL in the FE-ULBD group (*p* = 0.049) (Table 5). No other variables showed statistically significant associations. Given the relatively small sample size, multivariable regression analysis was not performed to avoid potential overfitting.

## 4. Discussion

Since the introduction of the concept of HBL in 2000, increasing attention has been directed toward its role in various spine surgeries [13,17,18,19]. Numerous studies have reported on HBL in microscopic and endoscopic procedures for lumbar disc herniation [4,12,20]. Notably, in these studies, full-endoscopic techniques primarily involved soft-tissue manipulation without bone resection. Despite growing evidence on HBL in ESS, the literature addressing HBL in full-endoscopic decompressive procedures involving bony work for LSS remains limited.

Although substantial evidence highlights the clinical relevance of HBL in spine surgery [18,21,22], attention in clinical practice often focuses on IBL and postoperative drainage volume rather than on HBL [12]. Previous studies have demonstrated that HBL can be considerable even in ESS, which is generally associated with lower perioperative blood loss compared with conventional open surgery [11,22,23]. In the present study, HBL accounted for a substantial proportion of TBL across all three groups, consistent with earlier reports. These findings indicate that surgeons should remain vigilant regarding HBL even in FESS, which shows the lowest perioperative blood loss among these surgical approaches.

Consistent with several previous studies reporting lower TBL with FESS compared with BESS and open microscopic techniques [4,12,20,24], our study also demonstrated significantly lower TBL in the FE-ULBD group than in the BE-ULBD and OM-ULBD groups. In BESS, detachment of paravertebral muscles from the lamina to create a working space for separate viewing and working portals may result in greater muscle injury. In contrast, FESS employs a muscle-splitting approach using serial dilators to access the target site, thereby minimizing muscle trauma. Moreover, whereas BESS requires a relatively large saline-maintained working space at the muscle–lamina interface [25], FESS is performed within a working sleeve, creating a more confined environment that may limit blood extravasation into surrounding soft tissues and potential dead spaces.

In the comparative analysis of HBL, the FE-ULBD group demonstrated significantly lower HBL than the BE-ULBD group in the post hoc analysis. However, post hoc analysis between the FE-ULBD and OM-ULBD groups did not demonstrate statistical significance, although median HBL was numerically lower in the FE-ULBD group. This may be attributable to the substantial variability and overlap in HBL distributions between the groups, particularly in the OM-ULBD group, which showed a wide IQR. In addition to the inherent difficulty of accurately measuring IBL in ESS, postoperative drainage volumes may be imprecise because of drainage of saline infiltrated during surgery. These factors may introduce error into the calculation of VBL and, consequently, HBL. Nevertheless, TBL—calculated using objective parameters including height, body weight, and perioperative hematocrit—was significantly lower in the FE-ULBD group than in both the BE-ULBD and OM-ULBD groups. Taken together, these findings suggest that FESS may still offer an advantage in reducing perioperative blood loss compared with BESS and conventional microscopic surgery. Further systematic studies using more precise methods for quantifying perioperative blood loss are warranted to better clarify differences in HBL among surgical techniques.

In the present study, male sex was identified as the only significant predictive factor for increased HBL in the FE-ULBD group. Several previous studies have also reported male sex as a significant factor associated with increased perioperative blood loss, possibly because male patients tend to have greater muscle mass [26,27]. However, the impact of sex on blood loss in ESS has not been consistently reported across the literature [3,4,20]. Therefore, further studies including larger numbers of patients are warranted to better clarify predictive factors associated with HBL in FE-ULBD.

Guo et al. suggested paraspinal muscle thickness at the index level as an independent risk factor for HBL in BESS [22]. Thicker paraspinal muscle requires longer working channels through which the endoscope and instruments pass, which may lead to increased intraoperative muscle bleeding and larger space for blood infiltration during surgery. In contrast, our results indicated that the paraspinal muscle thickness was not related to HBL in FE-ULBD. Unlike BESS and other types of spinal surgeries, procedures are performed within a working sleeve, and the working space does not communicate with the muscle layer. However, future research with a large sample size is needed to clarify the effects of soft-tissue thickness on HBL.

All three groups had favorable clinical outcomes, including VAS scores for radicular leg pain and modified Macnab criteria, without significant differences among groups in our study. Additionally, all groups demonstrated improved pain scores postoperatively compared to preoperatively. Previous studies have documented that various types of ESS have comparable postoperative clinical outcomes to open microscopic surgeries, with ESS showing lower immediate postoperative pain [5,8]. However, serial measurements of postoperative outcomes, including VAS scores for low back pain and immediate postoperative pain scores, were not consistently conducted in our study due to the retrospective design. Therefore, a well-designed prospective study comparing serial postoperative pain scores and clinical outcomes among FESS, BESS, and open microscopic surgeries is warranted.

In this study, the choice of surgical technique was determined by individual surgeons’ preference following discussion with the patients. As such, subjective preference may have been influenced by patient-specific parameters—including body weight, subcutaneous fat and muscle distribution, and degree of stenosis—which may have indirectly influenced perioperative blood loss. However, baseline comparisons across the three groups revealed no statistically significant differences in these parameters, suggesting that the groups were reasonably balanced with respect to factors most likely to confound blood loss outcomes. Nonetheless, the retrospective nature of this study precludes definitive conclusions, and well-controlled randomized studies with surgeries performed by a single surgeon are warranted to clarify the relationship between surgical technique selection and perioperative blood loss.

This study has several limitations. First, the retrospective design introduces the potential for selection bias and unmeasured confounding. Second, the relatively small sample size in each group limits the generalizability of the findings, and the present study should therefore be interpreted as exploratory in nature. Further studies with larger sample sizes and standardized blood loss measurement protocols are warranted to validate and extend these findings. Third, accurate measurement of IBL and postoperative drainage volume was challenging, which may have introduced measurement error into the calculations of HBL and VBL. Although the present study followed the methodology adopted in previously published blood loss studies in spine surgery, including the use of the Nadler and Gross formulas, future well-controlled studies with standardized blood loss measurement protocols are needed to more accurately quantify HBL. Fourth, the surgical technique was selected based on individual surgeons’ preference rather than well-controlled randomization. Although baseline characteristics were comparable across groups, the possibility of selection bias cannot be entirely excluded. Finally, only single-level ULBD procedures were included, and the findings may not be applicable to multilevel decompression or more complex surgical cases. Despite these limitations, to the best of our knowledge, this study is the first to evaluate HBL in lumbar decompressive surgery involving bony work by comparing FESS, BESS, and microscopic surgery.

## 5. Conclusions

This study demonstrated that FE-ULBD was associated with significantly lower TBL than both BE-ULBD and OM-ULBD, as well as significantly lower HBL than BE-ULBD, in single-level decompression for LSS. These findings suggest that FE-ULBD may represent a feasible surgical option in LSS, with the potential advantage of reduced perioperative blood loss while maintaining comparable clinical outcomes.

## Figures and Tables

**Table 1:** Comparison of baseline characteristics among groups.

	FE-ULBD (*n* = 34)	BE-ULBD (*n* = 32)	OM-ULBD (*n* = 27)	*p*-Value
Age (years)	67.56 ± 10.36	64.88 ± 11.74	69.63 ± 7.99	0.208
Sex, n (%)				0.718
Female	15 (44.1)	17 (53.1)	12 (44.4)	
Male	19 (55.9)	15 (46.9)	15 (55.6)	
Height (m)	1.62 ± 0.09	1.64 ± 0.09	1.62 ± 0.09	0.672
Weight (kg)	65.50 ± 11.94	68.39 ± 16.21	66.15 ± 8.71	0.638
BMI (kg/m^2^)	24.76 ± 3.43	25.23 ± 4.01	25.22 ± 2.59	0.819
Operative side, n (%)				0.094
Left	22 (64.7)	23 (71.9)	24 (88.9)	
Right	12 (35.3)	9 (28.1)	3 (11.1)	
Operative level, n (%)				0.193
L1–2	1 (2.9)	0 (0)	0 (0)	
L2–3	0 (0)	3 (9.4)	1 (3.7)	
L3–4	6 (17.6)	3 (9.4)	2 (7.4)	
L4–5	24 (70.6)	20 (62.5)	23 (85.2)	
L5–S1	3 (8.8)	6 (18.8)	1 (3.7)	
Degree of central canal stenosis, n (%)				0.718
Grade 2	20 (58.8)	21 (65.6)	15 (55.6)	
Grade 3	14 (41.2)	11 (34.4)	12 (44.4)	
Spondylolisthesis, n (%)	2 (5.9)	6 (18.8)	7 (25.9)	0.095
Liver dysfunction, n (%)	4 (12.1)	2 (6.3)	0 (0)	0.234
Antithrombotics, n (%)	7 (20.6)	9 (28.1)	11 (40.7)	0.225
ASA classification, n (%)				0.323
I	4 (11.8)	3 (9.4)	0 (0)	
II	27 (79.4)	27 (84.4)	25 (92.6)	
III	3 (8.8)	2 (6.3)	2 (7.4)	
Smoking, n (%)	8 (23.5)	6 (18.8)	1 (3.7)	0.099

Abbreviations: FE-ULBD, full-endoscopic unilateral laminotomy with bilateral decompression; BE-ULBD, biportal endoscopic ULBD; OM-ULBD, open microscopic ULBD; BMI, body mass index; ASA, American Society of Anesthesiologists physical status classification system score.

**Table 2:** Comparison of perioperative data among groups.

	FE-ULBD (*n* = 34)	BE-ULBD (*n* = 32)	OM-ULBD (*n* = 27)	*p*-Value
Operative time (min)	96.32 ± 13.27	83.28 ± 22.42	94.37 ± 32.12	0.057
Preoperative PLT (×10^3^/mm^3^)	226.38 ± 58.94	250.68 ± 88.83	228.96 ± 67.16	0.346
Preoperative PT (s)	12.71 ± 0.70	12.69 ± 0.78	12.96 ± 0.64	0.293
Preoperative PT-INR	0.97 ± 0.06	0.97 ± 0.08	0.99 ± 0.06	0.497
Preoperative APTT (s)	34.73 ± 3.10	33.03 ± 3.28	33.98 ± 2.91	0.089
Preoperative Hb (g/dL)	13.77 ± 1.50	13.52 ± 1.39	14.26 ± 1.29	0.132
Preoperative Hct (%)	40.60 ± 4.14	40.36 ± 3.91	42.51 ± 3.51	0.076
Postoperative Hb (g/dL)	12.11 ± 1.46	11.53 ± 1.32	11.71 ± 1.49	0.238
Postoperative Hct (%)	35.91 ± 3.54	34.29 ± 3.67	35.00 ± 4.25	0.227
Patients’ blood volume (L)	4.12 ± 0.73	4.21 ± 0.91	4.12 ± 0.65	0.853
Total blood loss (mL)	493.20 ± 183.46	675.97 ± 192.02	822.94 ± 424.11	<0.001 *
Visible blood loss (mL)	121.24 ± 80.12	143.13 ± 66.36	257.26 ± 166.03	0.001 *
Hidden blood loss (mL)	390.48 (268.32–506.91)	513.29 (437.96–633.36)	516.38 (316.41–710.68)	0.011 *^,^**
Total soft-tissue thickness (cm)	4.40 ± 0.96	4.84 ± 1.10	4.70 ± 0.72	0.167
Paraspinal muscle thickness (cm)	3.43 ± 0.61	3.44 ± 0.64	3.52 ± 0.45	0.783
Subcutaneous layer thickness (cm)	0.97 ± 0.73	1.38 ± 0.78	1.17 ± 0.60	0.065

Values are presented as mean ± standard deviation or median (interquartile range). Abbreviations: FE-ULBD, full-endoscopic unilateral laminotomy with bilateral decompression; BE-ULBD, biportal endoscopic ULBD; OM-ULBD, open microscopic ULBD; PLT, platelet; PT, prothrombin time; INR, international normalized ratio; APTT, activated partial thromboplastin time; Hb, hemoglobin; Hct, hematocrit. * Statistically significant (*p* < 0.05). ** Kruskal–Wallis test.

**Table 3:** Post hoc analysis of perioperative blood loss among groups.

	Mean Difference	Standard Error	*p*-Value
Total blood loss (mL)			
FE-ULBD vs. BE-ULBD	−182.77	68.35	0.001 *
FE-ULBD vs. OM-ULBD	−329.74	71.53	0.002 *
BE-ULBD vs. OM-ULBD	−146.97	72.51	0.234
Visible blood loss (mL)			
FE-ULBD vs. BE-ULBD	−21.89	26.79	0.694
FE-ULBD vs. OM-ULBD	−136.02	28.04	<0.001 *
BE-ULBD vs. OM-ULBD	−114.13	28.43	<0.001 *
Hidden blood loss (mL)			
FE-ULBD vs. BE-ULBD			0.012 *^,^**
FE-ULBD vs. OM-ULBD			0.081 **
BE-ULBD vs. OM-ULBD			1.000 **

Abbreviations: FE-ULBD, full-endoscopic unilateral laminotomy with bilateral decompression; BE-ULBD, biportal endoscopic ULBD; OM-ULBD, open microscopic ULBD. * Statistically significant (*p* < 0.05). ** Mann–Whitney U tests with Bonferroni correction.

**Table 4:** Comparison of clinical outcomes and complications among groups.

	FE-ULBD (*n* = 34)	BE-ULBD (*n* = 32)	OM-ULBD (*n* = 27)	*p*-Value
VAS—leg pain				0.151 *
Preoperative	8.12 ± 1.75	7.69 ± 1.12	7.81 ± 1.30	
Postoperative—3 months	2.71 ± 1.93	3.41 ± 1.86	3.30 ± 1.86	
Postoperative—6 months	3.12 ± 2.09	3.41 ± 2.41	3.74 ± 2.35	
Modified Macnab criteria, n (%)				0.344
Excellent + Good	30 (88.2)	25 (78.1)	20 (74.1)	
Fair + Poor	4 (11.8)	7 (21.9)	7 (25.9)	
Complications, n (%)	3 (8.8)	2 (6.3)	5 (18.5)	0.340
Dural tear	2 (5.9)	1 (3.1)	3 (11.1)	
Root injury	1 (2.9)	0 (0)	0 (0)	
Hematoma	0 (0)	1 (3.1)	1 (3.7)	
Wound problem	0 (0)	0 (0)	1 (3.7)	

Abbreviations: FE-ULBD, full-endoscopic unilateral laminotomy with bilateral decompression; BE-ULBD, biportal endoscopic ULBD; OM-ULBD, open microscopic ULBD; VAS, visual analogue scale. * Group-by-time interaction derived from repeated-measures analysis of variance.

**Table 5:** Univariate linear regression analysis of hidden blood loss in the FE-ULBD group.

	B (SE)	β	95% CI	*p*-Value	VIF
Sex (male)	138.04 (67.41)	0.340	0.74–275.35	0.049	1.000

Values are presented as unstandardized coefficients (B) with standard errors (SE), standardized coefficients (β), and 95% confidence interval (CI). Abbreviations: VIF, variance inflation factor.

## Data Availability

The data presented in this study are available on reasonable request from the corresponding author.

## Abbreviations

The following abbreviations are used in this manuscript:

ANOVA	Analysis of variance
BE-ULBD	Biportal endoscopic unilateral laminotomy with bilateral decompression
BESS	Biportal endoscopic spine surgery
ESS	Endoscopic spine surgery
FE-ULBD	Full-endoscopic unilateral laminotomy with bilateral decompression
FESS	Full-endoscopic spine surgery
HBL	Hidden blood loss
Hct	Hematocrit
IBL	Intraoperative blood loss
IQR	Interquartile range
LSS	Lumbar spinal stenosis
OM-ULBD	Open microscopic unilateral laminotomy with bilateral decompression
PBV	Patient blood volume
RM-ANOVA	Repeated-measures analysis of variance
TBL	Total blood loss
ULBD	Unilateral laminotomy with bilateral decompression
VAS	Visual analogue scale
VBL	Visible blood loss
